# Field ponding water exacerbates the dissemination of manure-derived antibiotic resistance genes from paddy soil to surrounding waterbodies

**DOI:** 10.3389/fmicb.2023.1135278

**Published:** 2023-03-16

**Authors:** Ming-Sha Zhang, Si-Zhou Liang, Wei-Guo Zhang, Ya-Jun Chang, Zhongfang Lei, Wen Li, Guo-Liang Zhang, Yan Gao

**Affiliations:** ^1^School of Resources and Environmental Sciences, Nanjing Agricultural University, Nanjing, China; ^2^Jiangsu Key Laboratory for the Research and Utilization of Plant Resources, Institute of Botany, Jiangsu Province and Chinese Academy of Sciences (Nanjing Botanical Garden Memorial Sun Yat-Sen), Nanjing, China; ^3^School of the Environment and Safety Engineering, Jiangsu University, Zhenjiang, China; ^4^School of Life and Environmental Sciences, University of Tsukuba, Tsukuba, Japan; ^5^Jiangsu Key Laboratory for Food Quality and Safety-State Key Laboratory Cultivation Base, Ministry of Science and Technology, Nanjing, China; ^6^School of Life Sciences, Nanjing University, Nanjing, China; ^7^Huaiyin Institute of Technology, Huaian, China; ^8^Institute of Agricultural Resources and Environment, Jiangsu Academy of Agricultural Sciences, Nanjing, China

**Keywords:** antibiotic resistance genes, field ponding water, rice paddy, manure fertilized soil, public health

## Abstract

Farmlands fertilized with livestock manure-derived amendments have become a hot topic in the dissemination of antibiotic resistance genes (ARGs). Field ponding water connects rice paddies with surrounding water bodies, such as reservoirs, rivers, and lakes. However, there is a knowledge gap in understanding whether and how manure-borne ARGs can be transferred from paddy soil into field ponding water. Our studies suggest that the manure-derived ARGs *aadA1*, *bla1*, *catA1*, *cmlA1-01*, *cmx(A)*, *ermB*, *mepA* and *tetPB-01* can easily be transferred into field ponding water from paddy soil. The bacterial phyla Crenarchaeota, Verrucomicrobia, Cyanobacteria, Choloroflexi, Acidobacteria, Firmicutes, Bacteroidetes, and Actinobacteria are potential hosts of ARGs. Opportunistic pathogens detected in both paddy soil and field ponding water showed robust correlations with ARGs. Network co-occurrence analysis showed that mobile genetic elements (MGEs) were strongly correlated with ARGs. Our findings highlight that manure-borne ARGs and antibiotic-resistant bacteria in paddy fields can conveniently disseminate to the surrounding waterbodies through field ponding water, posing a threat to public health. This study provides a new perspective for comprehensively assessing the risk posed by ARGs in paddy ecosystems.

## 1. Introduction

In recent years, the contamination of antibiotic resistance genes (ARGs) caused by misuse and overuse of antibiotics in healthcare and animal husbandry has attracted worldwide concern (Martínez, 2008; Zhu et al., 2013; Rodriguez-Mozaz et al., 2015; Zhang et al., 2022). Livestock manure is a hotspot that harbors abundant ARGs under selective pressure of antibiotics (Zhu et al., 2013; Jia et al., 2017; Congilosi and Aga, 2021; Macedo et al., 2022). The overuse of chemical fertilizers results in severe soil hardening, agricultural nonpoint source pollution, and disruption of the ecological balance (Jiao et al., 2017; Zou et al., 2020). Therefore, in recent years, some policies have been formulated and implemented to alleviate these problems. Of these, the well-known one is that “organic fertilizers partially replace chemical fertilizers” (Jiao et al., 2017; Zhang et al., 2021b). Consequently, amounts of livestock manure or manure-derived amendments as organic fertilizer have been poured into farmland, but the contamination of antibiotics and ARGs in farmland has become increasingly severe, attracting increasing attention (Ding et al., 2019; Pu et al., 2020; Zhang et al., 2022). Farmlands fertilized with manure or manure-derived amendments have become important sources of ARG contamination in other environments (Zheng et al., 2018; Zhang et al., 2021b). ARGs can be transferred from soil to vegetables and fruits, posing a direct threat to human health (Marti et al., 2013; Zhu et al., 2017; Mei et al., 2021; Zhang et al., 2022). The emergence and spread of antibiotic resistance kill approximately 4.95 million people worldwide annually (Ding et al., 2022).

Paddy fields are among the largest farmlands in the world. Compared with other farmlands, paddy fields are more convenient for transferring substances in the soil to other environments through field ponding water. Due to irrigation with reclaimed wastewater and fertilization with livestock manure amendments, the rice paddy fields have become a hotspot of antibiotics and ARGs (Awad et al., 2015; Zhou et al., 2019; Guo et al., 2021; Zhang et al., 2021b). Although the abundance, diversity and environmental behaviors of ARGs were well investigated in these studies, the interaction between the paddy soil and field ponding water was poorly understood. If ARGs can be transferred into field ponding water from paddy soil, then they will easily disseminate to the surrounding waterbodies, such as reservoirs, rivers, and lakes, some of which are important sources of drinking water (Zhang et al., 2021a). Consequently, the main objective of this study was to elucidate whether and how ARGs could be transferred into field ponding water from paddy soils fertilized with livestock manure. Microbial community is the key factor which determines the environmental behavior of ARGs (Hu et al., 2017; Guo et al., 2018; Zheng et al., 2018; Zhang et al., 2021a). Therefore, the relationship between the microbiome and resistome was investigated in our study. Further, to evaluate the direct threat of ARGs to human health, we assessed the correlation between ARGs and opportunistic pathogens detected in paddy soil and field ponding water. Horizontal gene transfer (HGT) plays an important role in ARG dissemination among different bacterial species (Aguila-Arcos et al., 2017; Lerminiaux and Cameron, 2019; Schmidt et al., 2022). Finally, the correlation between the mobile genetic elements (MGEs) and ARGs was determined.

## 2. Materials and method

### 2.1. Sampling site and sample collection

Soil was collected from a rice paddy field located at the Jiangsu Academy of Agricultural Sciences, Nanjing, China (32°01N, 118°52E). The agricultural soil in this area is classified as yellow-brown soil according to the Chinese Soil Taxonomy and as Udalf according to the US Soil Taxonomy. Commercial chicken manure was purchased from a farmer’s market in Nanjing. Fifty kilograms soil sample was mixed with 2.5 kg livestock manure thoroughly. This soil mixture was aged for 3 months from March 7, 2021 to June 6, 2021. In our study, six customized plastic pots were designed to conduct pot experiments (Supplementary Figure S1). Seven kilograms soil/manure mixture filled in each plastic pot. Six plastic pots were set as six repetitions. Sterile water without ARGs detection was used to steep the soil for 7 days. Eighteen healthy rice seedings with similar growth condition were selected to transplant. Before transplant, the roots of these rice seedings were washed with five times. The flushing water was collected. An inoculating loop was soaked in the flushing water, and then lined on the surface of solid plate of Luria-Bertani broth to detect the microbes. When bacteria were not detected, three rice seedings were transplanted in each pot. After transplant, the six repetitive plastic pots were placed in an artificial climate chamber ZRG-1000A (Binglin, China). Gas cylinders filled with sterile air were linked to an artificial climate chamber to continuously supply fresh air. This avoided contamination by airborne ARGs. During the experiment, the sterile water without ARGs detection was used to irrigate the soil keeping the water 5 cm above the soil surface. No organic or inorganic fertilizers were used during the whole experimental period. The temperature was kept at 30°C in daytime and 25°C in night. The photoperiod was kept 12 h form 6:00 A.M. to 18:00 P.M every day. The light intensity was 30,000 lux. Field ponding water and soil samples were collected on June 28, July 21, August 22, September 27, and October 22. Each time 200 ml of field ponding water was collected from each pot. The water samples were stored in plastic bottles and immediately transported to the laboratory and stored at 4°C. Nitrocellulose membranes (0.45 μm) were used to obtain the microorganisms through filtering the water sample. The obtained microbes were stored at −20°C. Each time 50 g of paddy soil from each pot was collected. After collection, the soil samples were immediately transported to the laboratory and stored at −20°C in Ziplock bags for extracting DNA. Eighteen ARGs and two MGEs were detected in the commercial chicken manure in this study (Supplementary Table S1). Among which, eight ARGs *aadA1*, *bla1*, *catA1*, *cmlA1-01*, *cmx(A)*, *ermB*, *mepA* and *tetPB-01*, and two MGE*s tnp-01* and -*02* were selected as target detecting genes. These eight manure-derived ARGs and two MGEs were not detected in the paddy soil before mixed with the chicken manure.

### 2.2. DNA extraction and real-time quantitative polymerase chain reaction (RT-qPCR)

DNA in paddy soil was extracted from 1.0 g of soil using the FastDNA Spin Kit for soil (MP Biomedical, United States), according to the manufacturer’s instructions. Field ponding water DNA was extracted using a PowerWater DNA isolation kit (Mobio, United States), according to the manufacturer’s instructions. The purity, quality, and quantity of extracted DNA were determined using the method described by Li et al. (2017). RT-qPCR was conducted using a CFX96 Touch Real-time PCR System (Bio-Rad, United States). The primers information of ARGs, MGEs, and 16S rRNA were shown in Supplementary Table S1. The mixtures were reacted in a 20 μl system. All qPCR reactions were conducted in triplicate for each primer set, including the non-template negative control. PCR amplification was conducted according to the following program: 95°C for 10 min to activate enzymes, followed by 40 cycles at 95°C for 30 s to denature DNA, then annealing at 60°C for 30 s. When the Ct value was less than 31 for more than two positive replicates, amplification was considered valid (Su et al., 2015). The absolute copy numbers of ARGs and MGEs were normalized to the absolute 16S rRNA gene copy numbers (Looft et al., 2012; Wang et al., 2014; Guo et al., 2018; Zhang et al., 2021b).

### 2.3. Amplicon high-throughput sequencing and data processing

To characterize bacterial communities, the V4 region of the 16S rRNA gene was amplified using the primer set 515F/806R (Bates et al., 2011). The low-quality reads were trimmed using CUTADAPT version 1.9.1. According to the unique barcodes, the sample data were separated from the reads. After removing the barcodes and primers, raw reads were generated. The chimeric sequences of raw reads were identified and removed using the UCHIME algorithm (Edgar et al., 2011) to yield effective tags (clean reads). UPARSE, version 7.0.1001 was used to cluster the clean sample reads. Sequences with a threshold similarity of 97% were binned into operational taxonomic units. The National Center for Biotechnology Information (NCBI) database was selected to deposit all 16S rRNA gene sequences generated in this study under the accession number PRJNA934052.

### 2.4. Data analysis and visualization

All mathematical operations were performed in Microsoft Office 365. The Sankey diagram was depicted with Origin 9.0. The co-occurrence network was analyzed and visualized using R software with the graph package. To detect significant correlations, we constructed a correlation matrix by calculating all possible pairwise Spearman’s rank correlations between the ARGs and bacterial taxa, ARGs and opportunistic pathogens, and ARGs and MGEs. The effective Spearman’s correlation coefficient (*ρ*) was set at > 0.8 on the premise of *p* < 0.05.

## 3. Results and discussion

### 3.1. Dynamic distribution of ARGs in rice paddy soil and field ponding water

**Figure 1:**
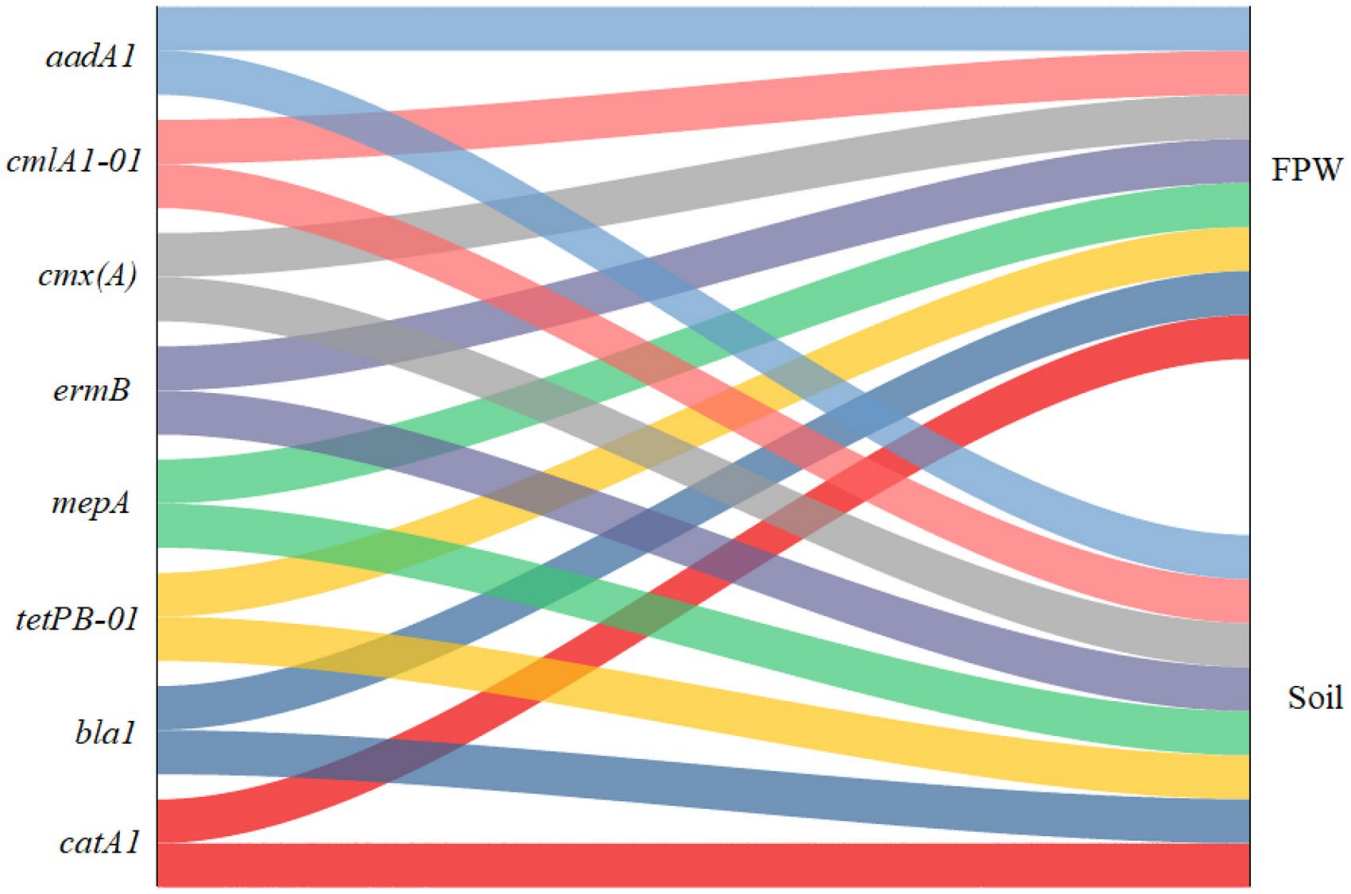
Sankey diagram depicting the distribution of antibiotic resistance genes (ARGs) in paddy soil fertilized with livestock manure and field ponding water (FPW).

Figure 1 shows that eight ARGs, *aadA1*, *bla1*, *catA1*, *cmlA1-01*, *cmx(A)*, *ermB*, *mepA*, and *tetPB-01*, were both detected in paddy soil and field ponding water. These genes confer resistance to aminoglycoside, beta-lactamase, MLSB (macrolide, lincosamide, and streptogramin B), multidrugs, and tetracycline. In addition, two MGEs, *tnp-01* and *-02*, were also detected in both paddy soil and field ponding water. These results indicate that ARGs can be easily transferred into field ponding water from paddy soils. Water bodies such as reservoirs, rivers, and lakes surrounded by paddy fields suffer from ARGs contamination (Yang et al., 2017; Wang et al., 2020; Zhang et al., 2021b). In these studies, the contamination sources mainly attributed to domestic sewage, livestock wastewater, aquaculture wastewater, and medical wastewater. Field ponding water is an important connection between paddy fields and surrounding waterbodies (Zhang et al., 2021a). Although the rice paddy fields have become a hotspot of antibiotics and ARGs because of irrigation with reclaimed wastewater and fertilization with livestock manure amendments, the rice paddy fields have become a hotspot of antibiotics and ARGs (Awad et al., 2015; Zhou et al., 2019; Guo et al., 2021; Zhang et al., 2021b), the interaction between the paddy soil and field ponding water has not been demonstrated yet. Our study verifies that field ponding water is also an important contamination source of ARGs to other large-scale water bodies, through facilitating the dissemination of manure-derived ARGs in paddy ecosystems.

**Figure 2:**
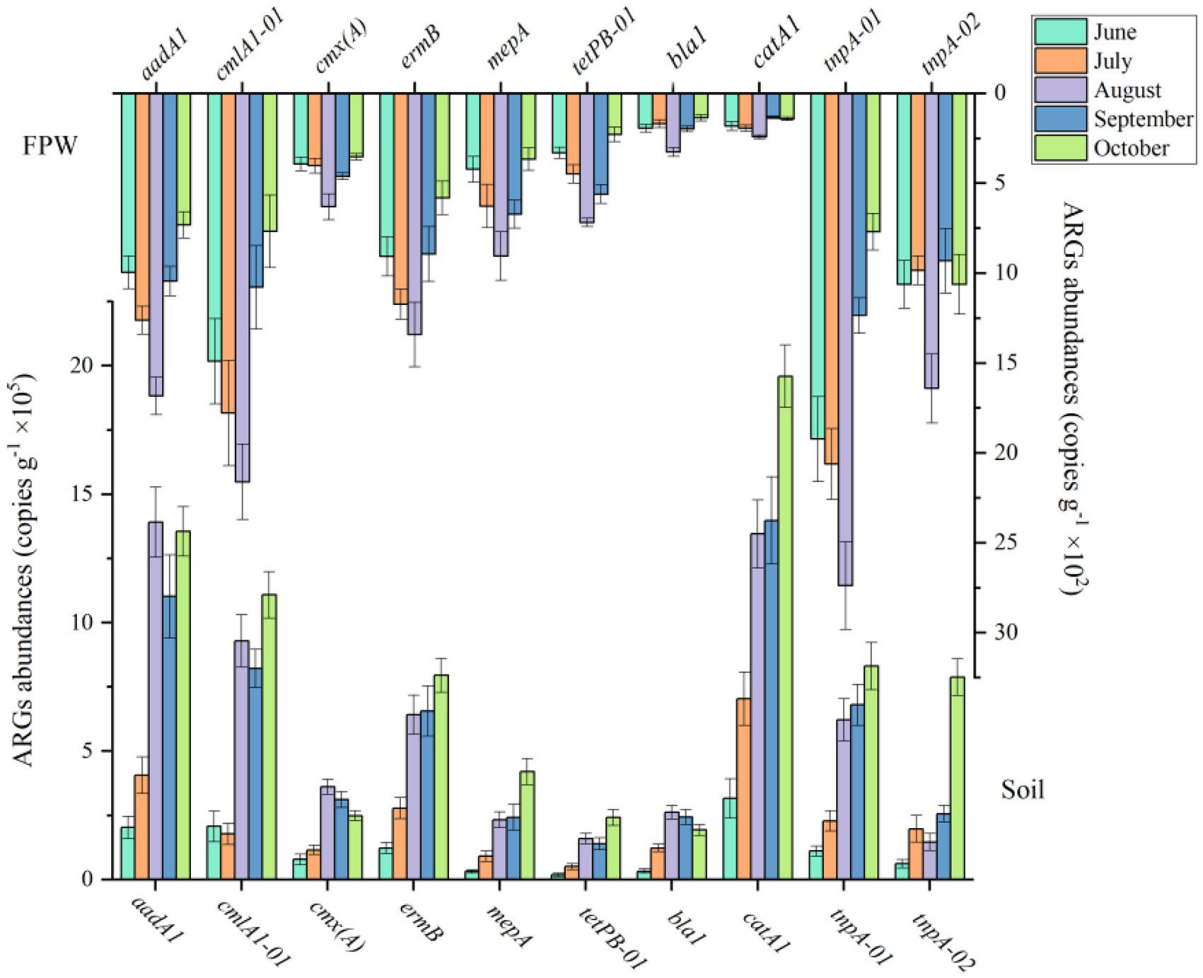
The dynamic abundances of ARGs in paddy soil fertilized with livestock manure amendment and field ponding water (FPW). The error bars represent ± SD (*n* = 6).

The abundance of ARGs in paddy soil was three orders of magnitude higher than that in field ponding water (Figure2). The paddy soil fertilized with chicken manure was the source of the antibiotic resistome in field ponding water. The number of genes in paddy soil increased from June to October. Specifically, the abundances of *aadA1*, *bla1*, *catA1*, *cmlA1-01*, *cmx(A)*, *ermB*, *mepA*, and *tetPB-01* in October were 5.68-, 5.03-, 5.22-, 4.35-, 2.18-, 5.49-, 12.27-, and 12.44-fold higher than those in June, respectively. Compared with other ARGs, the genes *catA1*, *aadA1* and *cmlA1-01* were more abundant in the paddy soil. The genes in field ponding water increased from June to August but decreased from August to October. The abundance of genes *cmlA1-01*, *aadA1* and *ermB* were higher than the other ARGs. The reasons for the two different tendencies of the antibiotic resistome in paddy soil and field ponding water are discussed in the following sections.

### 3.2. Microbial mechanisms driving the dynamic pattern of antibiotic resistome

**Figure 3:**
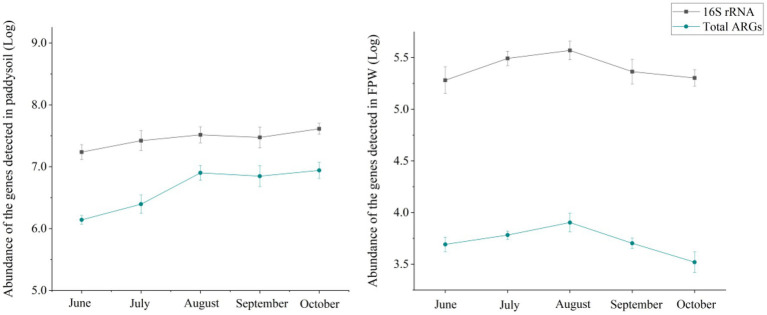
Abundances of 16S rRNA in paddy soil and field ponding water. The error bars represent ± SD (*n* = 6). FPW, field ponding water.

Although ARGs can exist in the environment in the form of eDNA, microbes are predominant in the prevalence of antibiotic resistome (Beaber et al., 2004; Martínez, 2008). The microbiome determines the pattern of antibiotic resistome (Hu et al., 2017; Guo et al., 2018; Zheng et al., 2018; Zhang et al., 2021a). In this study, the absolute abundances of 16S rRNA in paddy soil and field ponding water showed a similar tendency to that of the total ARGs (Figure 3).

**Figure 4:**
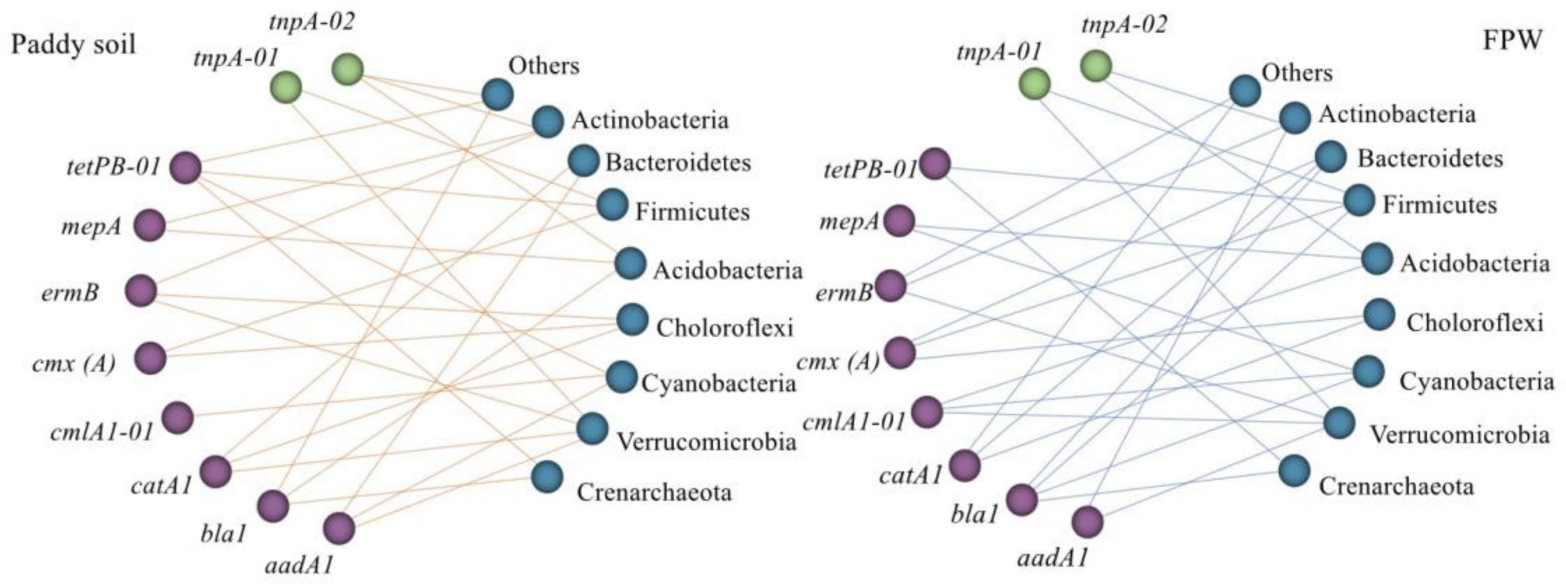
Correlation between ARGs and the major phyla. Spearman’s rank correlation was used to test the significance. The effective correlation coefficient (*ρ*) was set as > 0.8 on the premise of *p* < 0.05. Graphics were generated in R using the graph package. FPW, field ponding water.

The core bacterial taxa that intensively influenced the ARGs pattern have been also investigated in several previous studies through co-occurrence network analysis. Hu et al. (2017) found that the phyla Cyanobacteria, Actinobacteria, Gemmatimonadetes, and Crenarchaeota were the most likely ARG hosts in farmland with wheat and maize rotation. Wang et al. (2018) reported that the genera *Bradyrhizobium*, *Acidobacteria* Gp1, and *Gemmatimonas* were strongly correlated with ARGs in a dryland located in Jiangxi, China, whereas the genus *Acidobacteria* Gp2 was significantly correlated with ARGs in a paddy field. Wang et al. (2020) explored the relationship between the microbiome and antibiotic resistome in an agriculturally disturbed lake and found that the phyla Firmicutes, Gemmatimonadetes, Proteobacteria, and Verrucomicrobia exhibited a significantly positive correlation with ARGs. The results of Zhang et al. (2021a) showed that Acidobacteria, Actinobacteria, Chloroflexi, Firmicutes, Nitrospirae, and Verrucomicrobia were mainly attributed to the prevalence of ARGs in paddy fields located in Lake Tai Basin, China. These studies indicated that the core bacteria influencing the pattern of ARGs varied between biotopes. In this study, although the microbial alpha- and beta-diversity showed a significant variance between the paddy soil and field ponding water (Supplementary Figures S2, S3), the core bacterial taxa influencing the pattern of ARGs were similar in the paddy soil and field ponding water. The phyla Crenarchaeota, Verrucomicrobia, Cyanobacteria, Choloroflexi, Acidobacteria, Firmicutes, Bacteroidetes, and Actinobacteria had a significantly positive relationship with ARGs (Figure 4), indicating that these bacterial taxa exerted key roles in the environmental behaviors of these ARGs. Interestingly, the phylum Proteobacteria, the predomination microbial taxa in both paddy soil and field ponding water (Supplementary Figure S3), showed no significant correlation with ARGs. Similar phenomena were reported by Hu et al. (2017) and Zhang et al. (2021b), in which the predomination bacterial taxa also built weak correlation with ARGs while non-domination bacterial taxa showed robust correlations.

### 3.3. Correlation between ARGs and opportunistic pathogens

**Figure 5:**
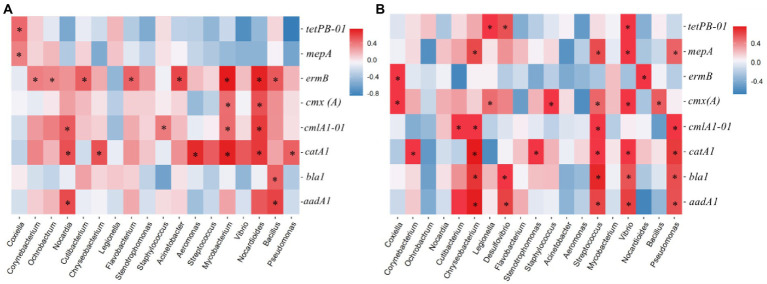
Heatmap showing the correlations between the opportunistic pathogens and ARGs detected in paddy soil **(A)** and field ponding water **(B)**. Spearman’s rank correlation was used to test the significance. ^*^p < 0.05.

Pathogens that carry ARGs pose a direct threat to human health (Sommer et al., 2009; Martínez et al., 2015; Crits-Christoph et al., 2022). If humans are infected with antibiotic-resistant pathogens, therapy will be intractable (Forsberg et al., 2012; Rajput et al., 2022). Numerous ARGs can be released into the environment, but not everyone having a higher risk. Recent studies proposed that the risk assessment of ARGs was decided based on three criteria: (1) enriched in human-related environments, (2) gene mobility, and (3) host pathogenicity (Zhang et al., 2021c; Yu et al., 2023). The genus which could be retrieved in the pathogenic bacteria database GlobalPPh and the previous references were identified as opportunistic pathogens. In this study, 18 opportunistic pathogens were identified in paddy soil and field ponding water. In paddy soil, *ermB*, *catA1*, *cmlA1-01*, *aadA1*, *cmx(A)*, *bla1*, *mepA*, and *tetPB-01* were positively correlated with eight, six, four, two, two, one, and one opportunistic pathogen, respectively; in field ponding water, *catA1*, *cmx(A)*, *aadA1*, *bla1*, *cmlA1-01*, *mepA*, *tetPB-01*, and *ermB* were positively correlated with six, six, five, five, four, four, three, and two opportunistic pathogens, respectively. These results suggest that field ponding water is an important pathway to disseminate potential antibiotic-resistant pathogens to the surrounding waterbodies (Figure 5).

### 3.4. Correlation between ARGs and MGEs

**Figure 6:**
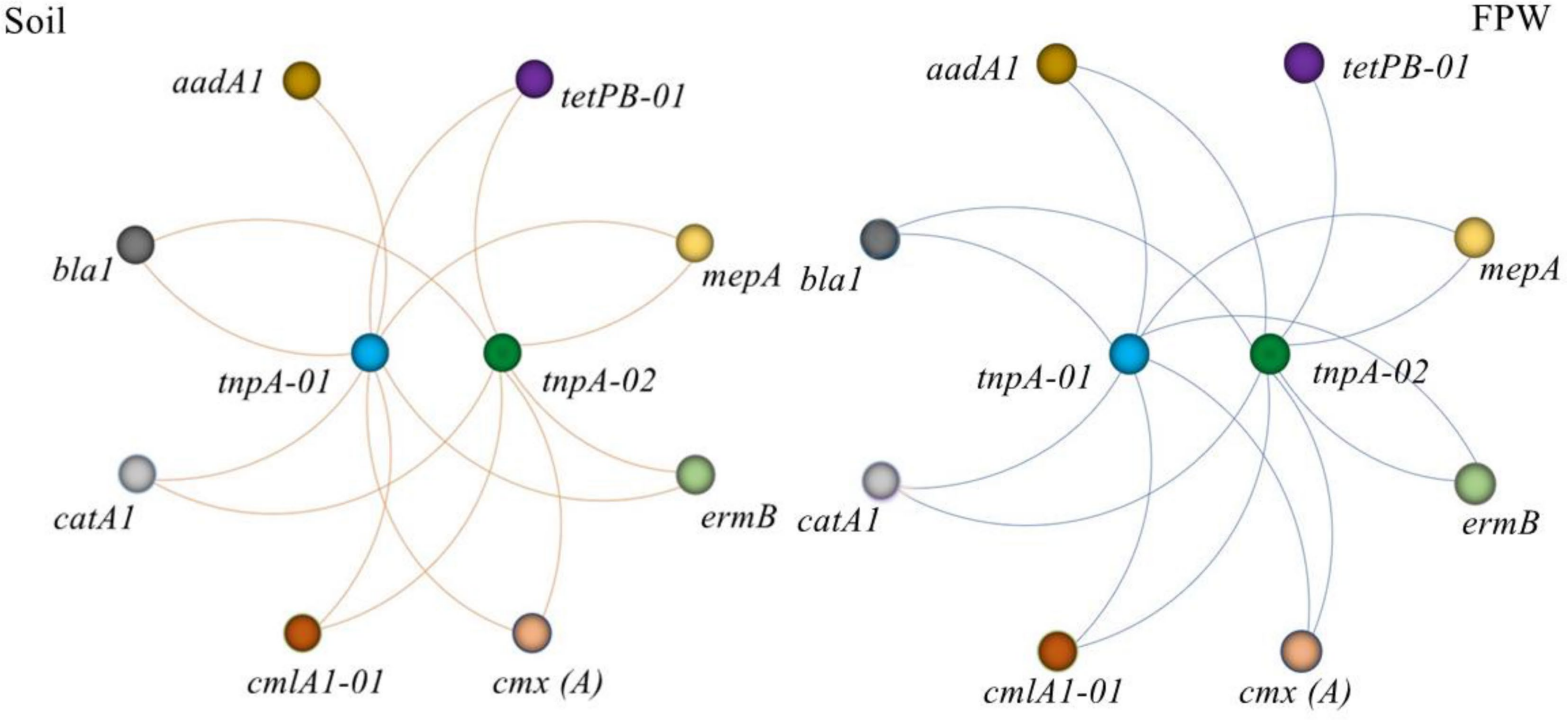
Network analysis depicting the correlations between antibiotic resistance genes and mobile genetic elements. Spearman’s rank correlation was used to test the significance. Each connection represents a significant Spearman’s correlation (*p* < 0.05). FPW, field ponding water.

Vertical gene transfer (VGT) and horizontal gene transfer (HGT) are two ways of disseminating ARGs in nature. Compared with VGT, the risk of HGT is higher because of its ability to transfer ARGs between different bacterial species (Gogarten and Townsend, 2005; Partridge et al., 2018; Abe et al., 2020; Lu et al., 2020; Li et al., 2022). The process of HGT is regulated by various of MGEs, including plasmids, transposons, and integrons (Zhu et al., 2013; Forsberg et al., 2014; Guo et al., 2017; Zhang et al., 2022). In this study, two manure-derived MGEs *tnp 01* and *02,* were detected in both paddy soil and field ponding water. As shown in Figure 6, all detected ARGs were significantly correlated with the two MGEs, indicating a potential dissemination ability of ARGs in both paddy soil and field ponding water. More importantly, once these antibiotic resistance bacteria went into the ponds, reservoirs, rivers and lakes *via* field ponding water, ARGs will be transferred to the protogenetic microbes, exacerbating the ARGs contamination.

## 4. Conclusion

In this study, the environmental behavior and driving mechanisms of ARGs in rice paddies fertilized with livestock manure were demonstrated. Our results highlighted that field ponding water can easily obtain ARGs from rice paddy soil, indicating that ARGs can conveniently disseminate to the surrounding waterbodies. The abundance of ARGs increased from June to October in the paddy soil; while that increased from June to August, but decreased from August to October. Microbiomes play a vital role in the dynamic pattern of antibiotic resistance. The phyla Crenarchaeota, Verrucomicrobia, Cyanobacteria, Choloroflexi, Acidobacteria, Firmicutes, Bacteroidetes, and Actinobacteria were potential ARG hosts. Eighteen opportunistic pathogens detected in both paddy soil and field ponding were significantly correlated with the corresponding ARGs. Network co-occurrence analysis between MGEs and ARGs in field ponding water suggests that ARGs have a strong dissemination ability across different bacterial species.

## Data availability statement

The raw data supporting the conclusions of this article will be made available by the authors, without undue reservation.

## Author contributions

W-GZ and YG designed the research. M-SZ, S-ZL, W-GZ, and WL conducted the lab work. W-GZ, M-SZ, and S-ZL performed the data analysis and wrote the manuscript in close consultation with all authors. G-LZ and Y-JC provided professional advice. ZL provided constructional advice in the revising process. All authors contributed to the article and approved the submitted version.

## Funding

This work was supported by the Jiangsu Agriculture Science and Technology Innovation Fund (CX(22)3001) and (CX(20)1010); Independent Research Foundation of Jiangsu Key Laboratory for Food Quality and Safety-State Key Laboratory Cultivation Base (2022-SBGJZZ-9).

## Conflict of interest

The authors declare that the research was conducted in the absence of any commercial or financial relationships that could be construed as a potential conflict of interest.

## Publisher’s note

All claims expressed in this article are solely those of the authors and do not necessarily represent those of their affiliated organizations, or those of the publisher, the editors and the reviewers. Any product that may be evaluated in this article, or claim that may be made by its manufacturer, is not guaranteed or endorsed by the publisher.
