# Supplementary material for: Field ponding water exacerbates the dissemination of manure-derived antibiotic resistance genes from paddy soil to surrounding waterbodies
Source: Front Microbiol. 2023 Mar 16;14:1135278. doi: 10.3389/fmicb.2023.1135278 (PMC10065064; doi:10.3389/fmicb.2023.1135278)
Supplement: Supplementary file 1 [file Data_Sheet_1.docx]

**Field ponding water exacerbates the dissemination of manure-derived antibiotic resistance genes from paddy soil to surrounding waterbodies**

Ming-Sha Zhang ^a,#^, Si-Zhou Liang ^b,c,#^, Wei-Guo Zhang ^a,c,d,e,⁎^, Ya-Jun Chang ^b,⁎^, Zhongfang Lei ^d^, Wen Li ^f^, Guo-Liang Zhang ^g^, Yan Gao ^h^

^a^ *School of Resources and Environmental Sciences, Nanjing Agricultural University, Nanjing 210095, China*

^b^ *Jiangsu Key Laboratory for the Research and Utilization of Plant Resources, Institute of Botany, Jiangsu Province and Chinese Academy of Sciences (Nanjing Botanical Garden Memorial Sun Yat-Sen), Nanjing 210014, China*

^c^ *School of the Environment and Safety Engineering, Jiangsu University, Zhenjiang 212013, China*

^d^ *School of Life and Environmental Sciences, University of Tsukuba, Tsukuba 305-8577, Japan*

^e^ *Jiangsu Key Laboratory for Food Quality and Safety-State Key Laboratory Cultivation Base , Ministry of Science and Technology, Nanjing 210014, China*

^f^ *School of Life Sciences, Nanjing University, Nanjing 210093, China*

^g^ *Huaiyin Institute of Technology, Huaian 223001, China*

^h^ *Institute of Agricultural Resources and Environment, Jiangsu Academy of Agricultural Sciences, Nanjing 210014, China*

^#^ These authors contributed equally to this article

^⁎^ Corresponding author, Wei-Guo Zhang, Institute of Agricultural Resources and Environment, Jiangsu Academy of Agricultural Sciences, Nanjing 210014, China. [weiguozhang@jaas.ac.cn](mailto:weiguozhang@jaas.ac.cn); Ya-Jun Chang, Jiangsu Key Laboratory for the Research and Utilization of Plant Resources, Institute of Botany, Jiangsu Province and Chinese Academy of Sciences (Nanjing Botanical Garden Memorial Sun Yat-Sen), Nanjing 210014, China. changyj@cnbg.net.


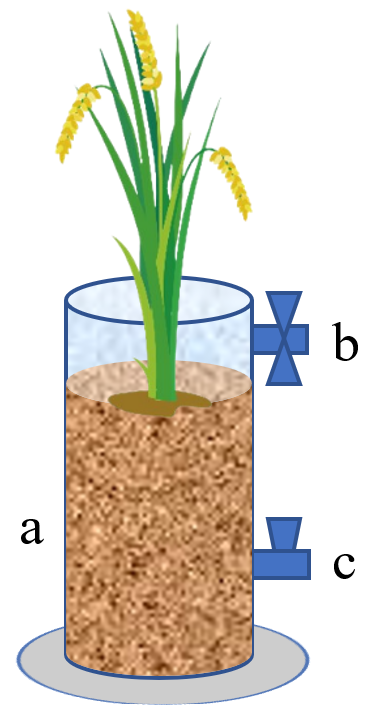


**Fig. S1.** The experimental equipment used to conduct pot experiments. The bottom area and depth of this equipment were 483 cm^2^ and 30 cm, respectively. a, plastic tube made from methyl methacrylate; b, the tap used to collect field ponding water; c, the tap used to collect paddy soil. When sampling the field ponding water, the tap b was opened. When sampling the paddy soil, the tap c was opened and a spoon with a long handle was used to stick into and grab the paddy soil.


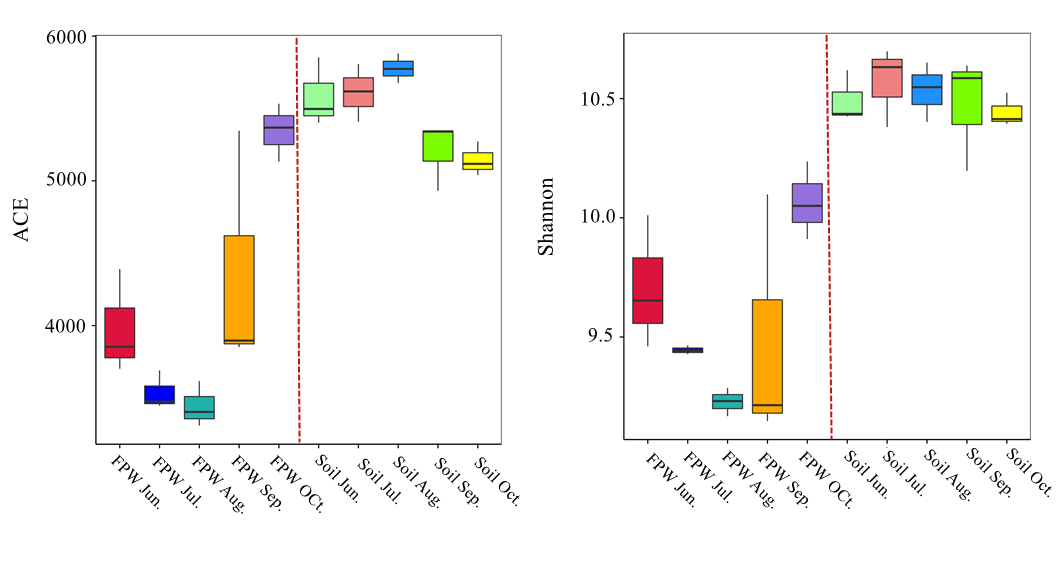
 **Fig. S2.** Alpha diversity (ACE and Shannon) of microbial community in paddy soil and field ponding water. FPW, field ponding water.


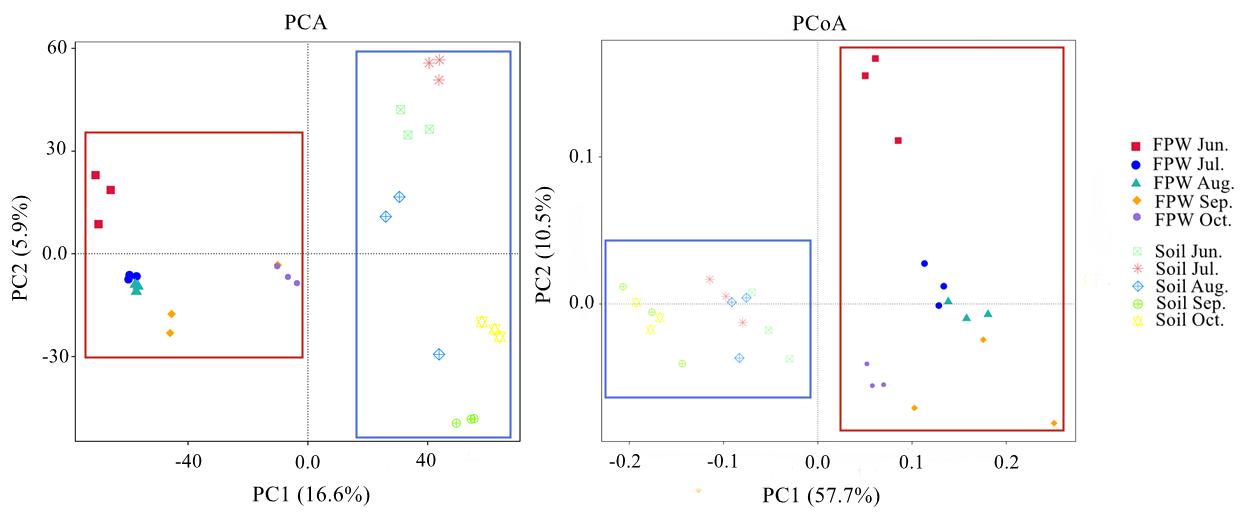


**Fig. S3.** The cluster analysis of microbial community (PCA and PCoA) basing on beta diversity indexes in paddy soil and field ponding water. FPW, field ponding water.

**Table S1.** Information of the ARGs detected in the commercial chicken manure and the corresponding primers. The primers of 16S rRNA was included in this table.

| **Number** | **Gene Name** | **Forward Primer** | **Reverse Primer** | **Classification** |
| --- | --- | --- | --- | --- |
| 1 | *16S rRNA* | GGGTTGCGCTCGTTGC | ATGGYTGTCGTCAGCTCGTG |  |
| 2 | *aac* | CCCTGCGTTGTGGCTATGT | TTGGCCACGCCAATCC | Aminoglycoside |
| 3 | *aadA1* | AGCTAAGCGCGAACTGCAAT | TGGCTCGAAGATACCTGCAA | Aminoglycoside |
| 4 | *aadE* | TACCTTATTGCCCTTGGAAGAGTTA | GGAACTATGTCCCTTTTAATTCTACAATCT | Aminoglycoside |
| 5 | *bla1* | GCAAGTTGAAGCGAAAGAAAAGA | TACCAGTATCAATCGCATATACACCTAA | Beta-Lactamase |
| 6 | *blaCMY* | CCGCGGCGAAATTAAGC | GCCACTGTTTGCCTGTCAGTT | Beta-Lactamase |
| 7 | *catA1* | GGGTGAGTTTCACCAGTTTTGATT | CACCTTGTCGCCTTGCGTATA | Chloramphenicol |
| 8 | *catB3* | GCACTCGATGCCTTCCAAAA | AGAGCCGATCCAAACGTCAT | Chloramphenicol |
| 9 | *cmlA1-01* | TAGGAAGCATCGGAACGTTGAT | CAGACCGAGCACGACTGTTG | Chloramphenicol |
| 10 | *cmx(A)* | GCGATCGCCATCCTCTGT | TCGACACGGAGCCTTGGT | Chloramphenicol |
| 11 | *ermB* | TAAAGGGCATTTAACGACGAAACT | TTTATACCTCTGTTTGTTAGGGAATTGAA | MLSB |
| 12 | *ermC* | TTTGAAATCGGCTCAGGAAAA | ATGGTCTATTTCAATGGCAGTTACG | MLSB |
| 13 | *ermF* | CAGCTTTGGTTGAACATTTACGAA | AAATTCCTAAAATCACAACCGACAA | MLSB |
| 14 | *mepA* | ATCGGTCGCTCTTCGTTCAC | ATAAATAGGATCGAGCTGCTGGAT | Multidrug |
| 15 | *mexF* | CCGCGAGAAGGCCAAGA | TTGAGTTCGGCGGTGATGA | Multidrug |
| 16 | *tetPB-01* | ACACCTGGACACGCTGATTTT | ACCGTCTAGAACGCGGAATG | Tetracycline |
| 17 | *tetT* | CCATATAGAGGTTCCACCAAATCC | TGACCCTATTGGTAGTGGTTCTATTG | Tetracycline |
| 18 | *tnpA-01* | CATCATCGGACGGACAGAATT | GTCGGAGATGTGGGTGTAGAAAGT | Transposase |
| 19 | *tnpA-02* | GGGCGGGTCGATTGAAA | GTGGGCGGGATCTGCTT | Transposase |
